# Supplementary material for: Increased Plasma Soluble PD-1 Concentration Correlates with Disease Progression in Patients with Cancer Treated with Anti-PD-1 Antibodies
Source: Biomedicines. 2021 Dec 16;9(12):1929. doi: 10.3390/biomedicines9121929 (PMC8698555; doi:10.3390/biomedicines9121929)
Supplement: Supplementary file 1 [file biomedicines-09-01929-s001.zip › biomedicines-146406 supplementary Figure S1.pdf]

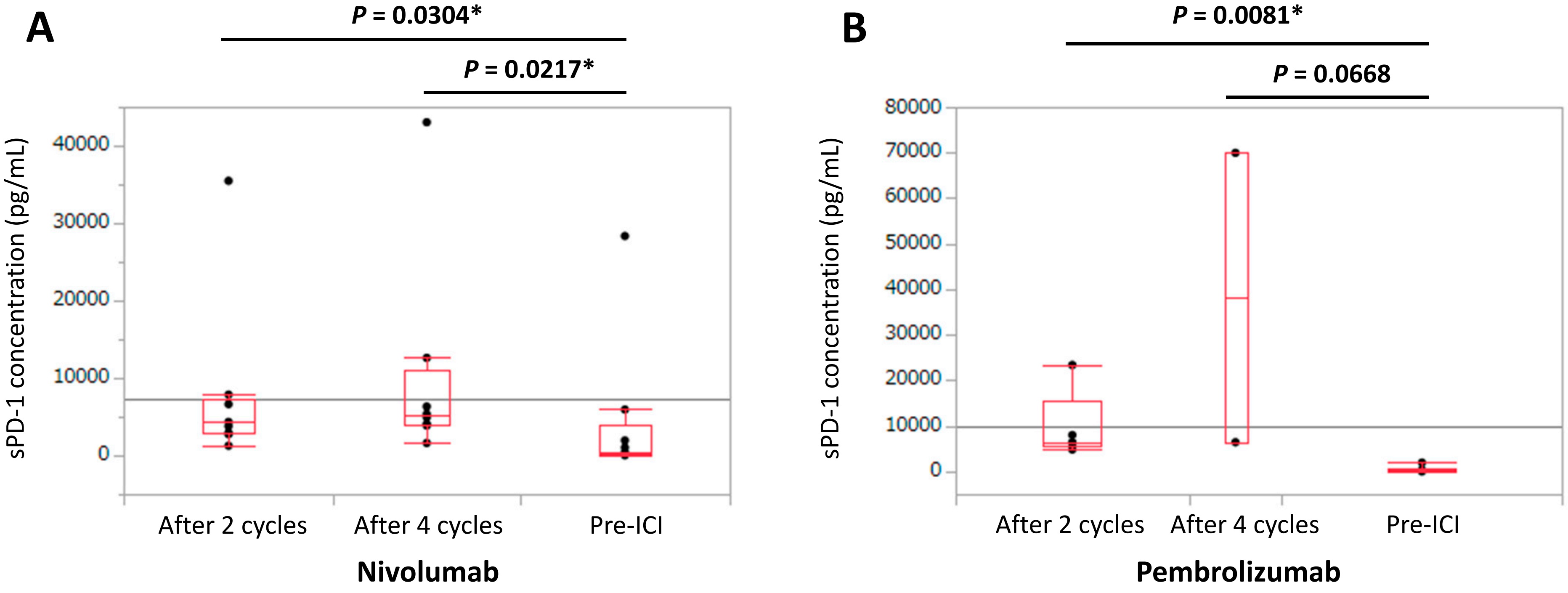

Figure S1. Comparison of sPD-1 levels between nivolumab and pembrolizumab group. (A) The sPD-1 concentrations in nivolumab group were compared at Pre-ICI, after 2 cycles, and after 4 cycles. **(B)** The same analysis was conducted for pembrolizumab group. *Statistically significant.
