# Supplementary material for: Increased Plasma Soluble PD-1 Concentration Correlates with Disease Progression in Patients with Cancer Treated with Anti-PD-1 Antibodies
Source: Biomedicines. 2021 Dec 16;9(12):1929. doi: 10.3390/biomedicines9121929 (PMC8698555; doi:10.3390/biomedicines9121929)
Supplement: Supplementary file 1 [file biomedicines-09-01929-s001.zip › biomedicines-146406 supplementary Figure S2.pdf]

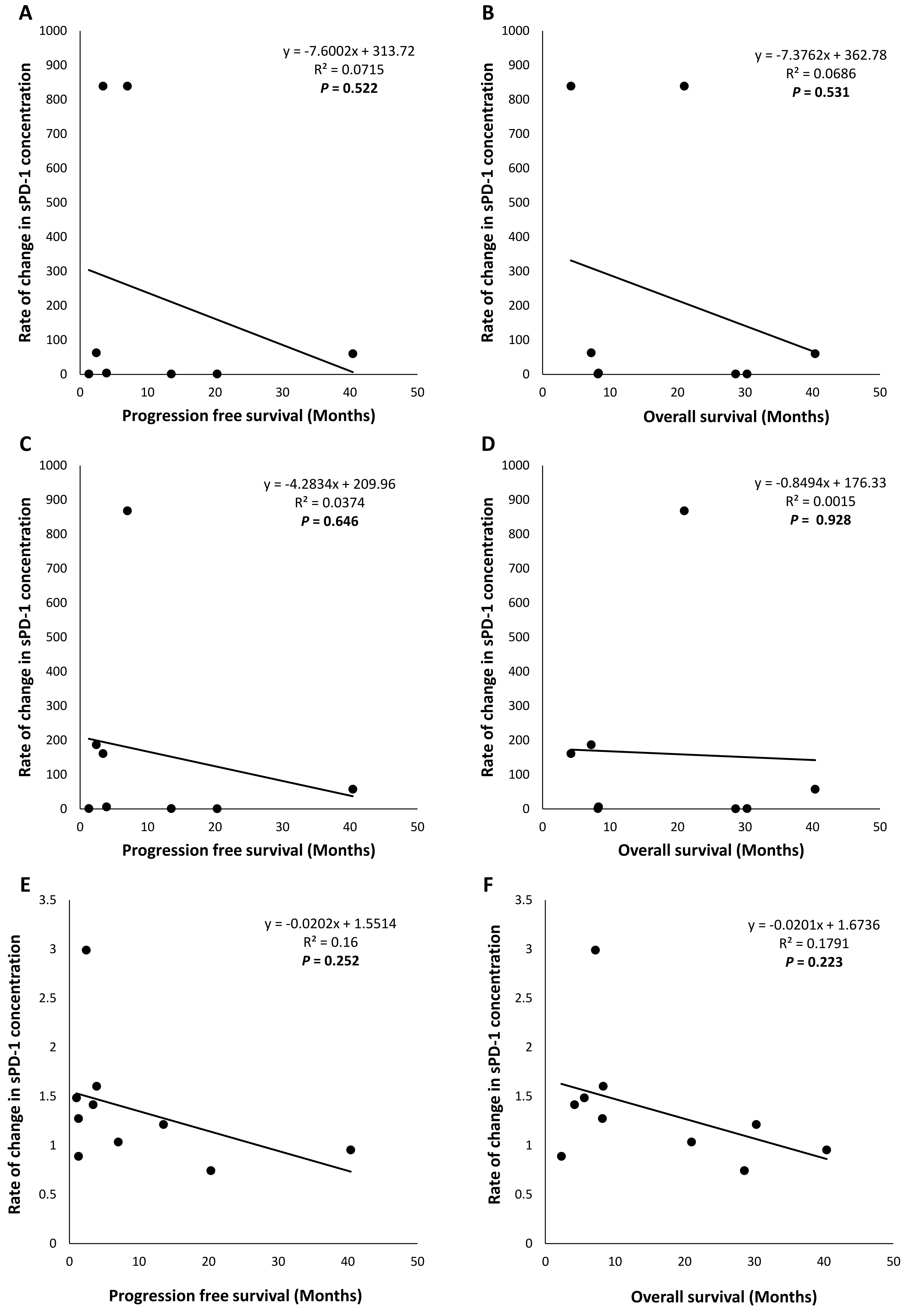

Figure S2. Linear correlation between change in plasma level of sPD-1 and PFS/OS. Relative changes in sPD-1 were calculated as the concentration from the baseline (pre-ICI treatment) to after two and four cycles and two to four cycles of ICIs therapy. Then, we analyzed the correlation between changes in sPD-1 levels and PFS/OS. There was no statistical association comparing among sPD-1 concentrations of pre-ICI, after 2 cycles, and after 4 cycles. (A) We analyzed the correlation between changes in sPD-1 levels from the baseline to after 2 cycles and PFS (r = 0.0715, p = 0.522), (B) from the baseline to after 2 cycles, and OS (r = 0.0686, p = 0.531). (C) We analyzed the correlation between changes in sPD-1 levels from the baseline to after 4 cycles and PFS (r = 0.0374, p = 0.646), (D) from baseline to after 4 cycles and OS (r = 0.0.0015, p = 0.928). (E) We analyzed the correlation between changes in sPD-1 levels from after 2 cycles to after 4 cycles and PFS (r = 0.16, p = 0.252), (F) from after 2 cycles to after 4 cycles and OS (r = 0.1791, p = 0.223).
